# Serum C-reactive protein is an important and powerful prognostic biomarker in most adult solid tumors

**DOI:** 10.1371/journal.pone.0202555

**Published:** 2018-08-23

**Authors:** Shiva Shrotriya, Declan Walsh, Amy S. Nowacki, Cliona Lorton, Aynur Aktas, Barbara Hullihen, Nabila Benanni-Baiti, Katherine Hauser, Serkan Ayvaz, Bassam Estfan

**Affiliations:** 1 Section of Palliative Medicine and Supportive Oncology, Department of Solid Tumor Oncology, Cleveland Clinic Taussig Cancer Institute, Cleveland, Ohio, United States of America; 2 The Harry R. Horvitz Center for Palliative Medicine, Cleveland Clinic Taussig Cancer Institute, Cleveland, Ohio, United States of America; 3 Department of Internal Medicine, Michigan State University, East Lansing, Michigan, United States of America; 4 Faculty of Health Sciences, Trinity College, Dublin, Ireland, United Kingdom; 5 School of Medicine and Medical Science, University College Dublin, Dublin, Ireland, United Kingdom; 6 Department of Quantitative Health Sciences, Cleveland Clinic, Cleveland, Ohio, United States of America; 7 ITD Analytics eResearch Department, Cleveland Clinic, Cleveland, Ohio, United States of America; University of South Alabama Mitchell Cancer Institute, UNITED STATES

## Abstract

**Introduction:**

Prognostication in cancer is challenging and inaccurate. C-Reactive Protein (CRP), a cheap and sensitive marker of inflammation may help. This study investigated the relationship between CRP and prognosis in a large cohort of solid tumors with mixed cancer diagnoses and stages.

**Methods:**

Electronic medical records of 4931 adults with solid tumors who attended the Taussig Cancer Institute from 2006–2012 were reviewed. Demographic and clinical characteristics were recorded. Maximum CRP (mCRP) was identified for each individual. CRP was analysed as a time-dependent, continuous and categorical variable for association with survival.

**Results:**

Two thirds of patients had a high mCRP. This was consistently associated with shorter survival, even after correction for time from diagnosis, and when analysed as a continuous or a categorical variable. When mCRP values above 10 mg/L were subcategorized, a higher mCRP was always worse. Even among those with normal values, statistically and clinically significant shorter survival was noted at mCRP levels >5 mg/L.

**Conclusions:**

In a large representative cohort of consecutive solid tumor patients the risk of death was clinically and statistically significantly greater with a high mCRP. This was independent of other variables and regardless of statistical method from both dates of diagnosis and test. CRP appeared to be underutilized. Our results support the routine use of CRP as a universal cost-effective independent prognostic indicator in most solid tumors.

## Introduction

Prognostication in cancer is still an inexact science. Physicians are often inaccurate and overly optimistic [1]. Clinical characteristics (lymph node status, male gender, performance status, tumor size) and some biomarkers (alfa-fetoprotein, lactate dehydrogenase) are helpful in certain tumors. Other established biomarkers (e.g. carbohydrate antigen 19–9, carcinoembryonic antigen, prostate specific antigen) are only valuable in specific tumors. Some have been clinically validated [2]. A more widely applicable prognostic biomarker is needed. A few biologic compounds meet the criteria for an ideal tumor marker; C-reactive protein (CRP) is one [3]. It is a non-specific acute phase reactant which reflects tissue damage. Serum concentration depends upon synthesis rate. Serum CRP is a sensitive and stable marker of inflammation. It can be measured by simple inexpensive methods [4].

Hepatocyte CRP secretion is controlled by interleukin 6 (IL-6). Interleukin-1 (IL-1) and tumor necrosis factor (TNF) also stimulate synthesis. A rise in serum levels often reflects the intensity of various pathological processes [5]. High serum CRP is also associated with a greater risk of cardiovascular events. In some chronic inflammatory diseases e.g. rheumatoid arthritis, serial levels correlate with both disease severity and therapeutic response.

There is some evidence to support the role of CRP as a prognostic indicator in specific primary sites [6, 7] and advanced disease [8]. Baseline CRP predicted mortality in operable lung cancer. [9]. In pancreatic cancer CRP, poor functional ability and rate of weight loss all increased near death [10]. CRP may also predict post-surgical tumor recurrence therapeutic response and toxicity [11]. Elevated high sensitivity (hs-CRP) levels have been associated with increased mortality in breast, lung and renal cell carcinomas [12]. Despite these observations CRP seems underused as a biomarker in routine oncology practice. It has been included in some cancer prognostic scoring systems [13]. However, in most reports of CRP and cancer prognosis, survival was not the primary study objective [14]. We decided to investigate the relationship between CRP level and prognosis utilizing the electronic medical records of 4931 persons with solid tumors who had CRP measured subsequent to their cancer diagnosis.

## Methods

### Study aims

1. Are high serum CRP levels predictive of prognosis in solid tumors?
2. Do CRP levels correlate with other known clinical characteristics (comorbidities, metastatic disease, treatment modalities or laboratory values)?
3. Are specific solid tumors associated with higher CRP levels?

### Study design

This retrospective cohort study utilized clinical data from an electronic medical record (EMR) (My Practice/EPIC, 1979–2014 Epic Systems Corporation, WI, USA). The protocol was approved by the Cleveland Clinic Institutional Review Board (IRB). Waiver of informed consent was granted. Adults who attended the Taussig Cancer Institute from 2006–2012 with a solid tumor diagnosis identified by International Classification of Disease Codes Version 9 (ICD-9, World Health Organization 2008) and with at least one serum CRP measurement and at least one total white blood cell count (TWBC) post-diagnosis were included.

People with hematological malignancies or without a TWBC and those whose CRP and/or TWBC measurements preceded the cancer diagnosis were excluded. Those with only one CRP value and a concurrent high TWBC were excluded to avoid co-morbid inflammatory illnesses or intercurrent procedures which may have temporarily elevated CRP. All CRP tests were performed with turbidimetric immunoassay (Roche Kobas, North America) at the Department of Pathology in the Cleveland Clinic.

### C-reactive protein

Serum CRP level was the primary measure. Due to the observational nature of the study, CRP had not been measured at consistent time points. A value >10 mg/L is a clinically accepted (though biologically unproven) cutoff-point of CRP into high versus normal groups. Maximum CRP (mCRP) was initially chosen for analysis as it likely reflects peak inflammatory status. In clinical practice it is impossible to identify the mCRP prospectively since this requires all values from diagnosis to death. mCRP years or decades before death may not adequately represent the actual level at death. CRP levels were therefore evaluated in three ways:

- mCRP post-diagnosis as a continuous variable
- mCRP post-diagnosis categorized as normal (maximum CRP ≤10 mg/L) and high
- CRP as a time-dependent variable: this took into account time from diagnosis.

### Data

These included: age, race, gender, primary cancer site(s), primary sites (number), metastatic sites (number), liver metastases (yes/no), co-morbidities (heart, liver or inflammatory bowel disease, rheumatoid arthritis), thromboembolic events (superficial or deep vein thrombosis or pulmonary embolism), possible cancer-related symptoms recorded (anorexia, cachexia, delirium, dysphagia, fatigue, malaise, pain, early satiety, weight loss), serum CRP (mg/L), total white blood cell (TWBC) count (k/μL), albumin (g/dL), hemoglobin (g/dL), body mass index (BMI) (Underweight <18.5; Normal 18.5–24.9; Overweight 25–29.9; Obese >30) [15], therapeutic interventions (anti-neoplastic chemotherapy, aspirin, biologic therapies, corticosteroids, hormonal therapy, non-steroidal anti-inflammatory drugs, radiotherapy, statins) and any invasive procedures (biopsies, stents, surgeries) within the four weeks before the mCRP test date. Only (clinical or laboratory) data from the same day or within 4 weeks prior to the mCRP were included. The exception was post-diagnosis mTWBC. TWBC was treated both as a time-dependent and a continuous variable (maximum TWBC). The modified Glasgow Prognostic Score (mGPS) (score 0–2) was also calculated: normal mCRP (≤10 mg/L) = 0; high mCRP (>10 mg/L) plus albumin (≥ 35 g/L) = 1; high mCRP (>10 mg/L) plus hypoalbuminemia (<35 g/L) = 2.

### Statistical analyses

Survival time was measured from two index dates; time to death from cancer diagnosis and time to death from date of mCRP. The former is clinically relevant. The latter is biologically relevant as it represents the time from assumed peak inflammatory state: it is less helpful clinically as the mCRP value date is only known retrospectively. Date of death was retrieved either from the Social Security Death Index (United States Social Security Administration) or the electronic medical record (EMR). Those alive at the last visit date were censored. This date was defined as either the last visit to the Cancer Institute (for any purpose) or the final laboratory measurement on record (last CRP; last TWBC).

Data was reported with descriptive statistics: frequencies (percentages) for categorical variables, mean +/- standard deviation or median (interquartile range) for continuous variables. Normal and high mCRP groups were compared with respect to continuous variables by independent t-tests and categorical variables by Fisher’s exact tests. Kaplan-Meier survival curves were generated to compare mCRP groups with log-rank tests. For subgroup analysis of mCRP values, groups were created based solely on frequencies and blinded to survival. Cox proportional hazards regression was performed to obtain hazard ratios (HR). HR quantified associations among clinical factors (e.g. mCRP, TWBC, mGPS) and mortality. Regression models were both adjusted and unadjusted for multiple variables. All percentages were rounded off to the nearest whole number. Analyses were performed with SAS (version 9.3) and JMP Pro (version 9) statistical software (SAS Institute Inc., Cary, NC, USA). P-values ≤ 0.05 (two sided) were considered statistically significant.

Prognosis was analyzed by mCRP group. A significant survival difference was observed between the normal and high groups both from the date of cancer diagnosis and mCRP test date. A similar survival analysis was done from mCRP test date. In both adjusted and unadjusted models, higher levels were associated with shorter survival both from date of diagnosis and test date.

**Fig 1:**
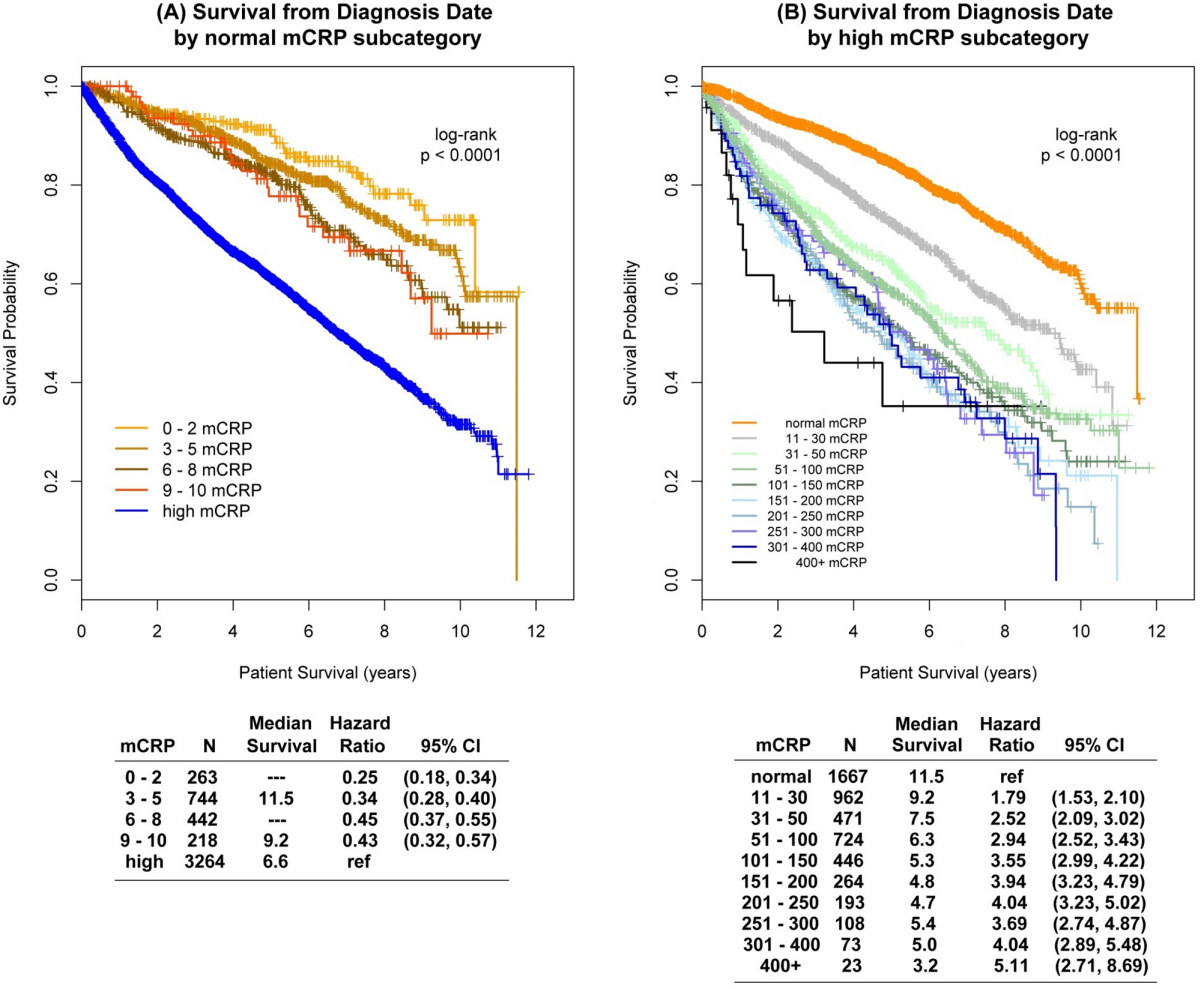
Prognosis by maximum CRP subgroup.

To further elucidate the mCRP association with survival, both the normal and high mCRP groups were subcategorized (Fig 1). In general, survival times were shorter in successive categories with higher mCRP values in both groups. When stratified by primary cancer site, higher mCRP values were associated with increased risk of death, regardless of tumor site. When mCRP was categorized the hazard ratio for high compared to normal mCRP ranged from 1.35 to 7.37.

## Results

7716 patients attended the Taussig Cancer Institute from 2006–2012 with a solid tumor diagnosis. The total study population was representative of the US population by gender (49% male) and race (83% Caucasians, 13% African Americans). The study cohort (N = 4931) was also representative of cancer mortality in the United States by tumor site prevalence. Their median length of follow-up was 4.0 years (range 0–11.8). The median number of CRP measurements per patient was 1 (range 1–87) and for TWBC 2 (range 1–96).

**Fig 2:**
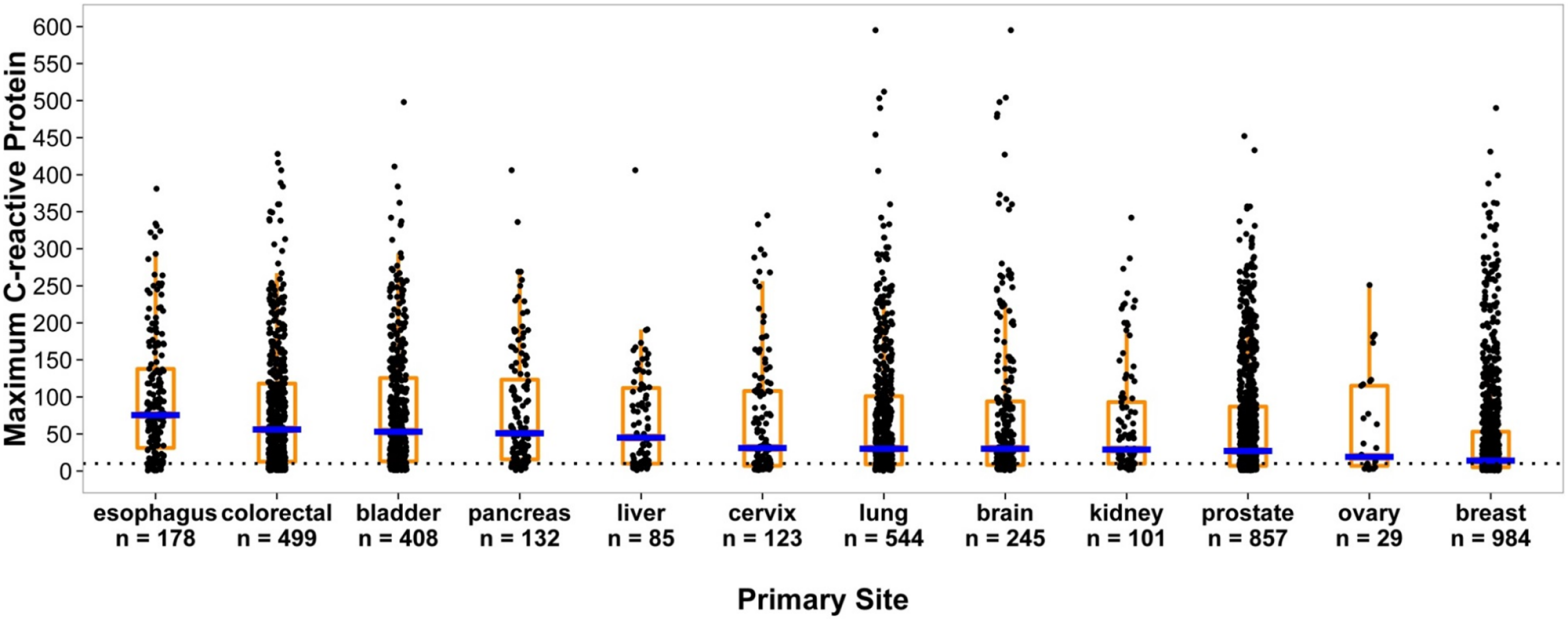
Maximum CRP value by primary cancer site.

4931 (63%) met all inclusion/exclusion criteria (Table 1). Table 2 compares the normal (34%, n = 1667) and high (66%, n = 3264) mCRP groups. The two groups were similar with respect to age, race, comorbidities and BMI. The normal group median (Q_1_—Q_3_) mCRP value was 5 (3–7) mg/L and the high group 62 (26–130) mg/L. More of the high group were male (53% vs. 41%), with a greater number of metastatic sites, and more often had surgery (31% vs. 8%) and stent insertions (2% v 1%) post-cancer diagnosis. Serum albumin and plasma hemoglobin levels were lower and maximum TWBC greater in the high mCRP group. Primary cancer site(s) were identified for each patient. mCRP values were higher in certain solid tumor primary sites (Fig 2). The highest median mCRP values = mg/L in descending order were: esophagus = 76, colorectal = 56, bladder = 53, pancreas = 51, liver = 45, cervix = 31, lung = 30, brain = 30, kidney = 29, prostate = 27, ovary = 19, and breast = 14. The association of mCRP and survival by primary cancer type was statistically significant for all sites.

**Table 1:** Patient demographic characteristics.

Variable	Solid Tumor Cancer Patients n = 4931
Age at Diagnosis (years)*	64 ± 14
Male	2415 (49%)
Primary Cancer Site*	
Breast	984 (20%)
Prostate	857 (17%)
Lung	544 (11%)
Colorectal	499 (10%)
Bladder	408 (8%)
Brain	245 (5%)
Esophagus	178 (4%)
Pancreas	132 (3%)
Cervix	123 (3%)
Kidney	101 (2%)
Liver	85 (2%)
Ovary	29 (0.5%)
Metastatic sites	
0	3667 (74%)
1	727 (15%)
2	295 (6%)
3+	242 (5%)

mean ± standard deviation or count (%)

* few patients had multiple primary sites

**Table 2:** Demographic and clinical variables in solid tumors by maximum CRP status.

	Normal mCRP (≤ 10 mg/L)	High mCRP (> 10 mg/L)	p-value**
Count	1667 (34%)	3264 (66%)	
Age at Diagnosis (years)	64 ± 13	65 ± 14	0.06
Male	685 (41%)	1730 (53%)	< .0001
Body Mass Index, BMI*	28.0 (24.6–32.4)	27.0 (23.5–31.6)	0.0001
Metastatic Sites			< .0001
0	1399 (84%)	2268 (69%)	
1	164 (10%)	563 (17%)	
2	52 (3%)	243 (7%)	
3+	52 (3%)	190 (6%)	
Liver metastases	64 (4%)	284 (9%)	< .0001
Comorbidities			
Cardiovascular inflammatory disease	220 (13%)	628 (19%)	< .0001
Inflammatory bowel disease	40 (2%)	84 (3%)	0.77
Liver disease	81 (5%)	265 (8%)	< .0001
Rheumatoid arthritis	127 (8%)	230 (7%)	0.49
Venous thromboembolism	178 (11%)	613 (19%)	< .0001
Treatment			
Aspirin	460 (28%)	890 (27%)	0.81
Biologic therapy	14 (1%)	55 (2%)	0.02
Chemotherapy	367 (22%)	616 (19%)	0.01
Corticosteroid	258 (15%)	719 (22%)	< .0001
Hormonal therapy	179 (11%)	283 (9%)	0.02
Non-steroidal anti-inflammatory	397 (24%)	499 (15%)	< .0001
Statin	588 (35%)	1063 (33%)	0.06
Surgery after Cancer Diagnosis	132 (8%)	1008 (31%)	< .0001
Stent after Cancer Diagnosis	11 (1%)	57 (2%)	0.002
Maximum TWBC, k/uL*	7.1 (5.9–8.4)	8.8 (7.0–11.2)	< .0001
Albumin, g/dL*	4.1 (3.8–4.4)	3.2 (2.6–3.9)	< .0001
Hemoglobin, g/dL*	13.2 (12.1–14.2)	11.0 (9.5–12.6)	< .0001
Modified Glasgow Prognostic Score (mGPS)			< .0001
0: CRP ≤ 10 mg/L	1667 (100%)	0 (0%)	
1: CRP > 10 mg/L & Albumin ≥ 35 gm/L	0 (0%)	1130 (42%)	
2: CRP > 10 mg/L & Albumin < 35 gm/L	0 (0%)	1559 (58%)	

*mean ± standard deviation, median (Q1—Q3) or count (%)

** independent t test or Fisher's exact test

CRP was analyzed in three ways as a continuous, categorical, and time-dependent variable. Regardless of whether CRP or mCRP were used, higher levels were associated with increased mortality where survival was time from cancer diagnosis to death. Unadjusted and adjusted regression models evaluated this association (Table 3). For CRP analyzed as a time-dependent continuous variable, for every ten unit rise (10 mg/L) the risk of death increased 3% (HR 1.003; 95% CI 1.003–1.004; p < .0001) when controlled for all other model variables.

**Table 3:** CRP and prognosis.

	**Univariable**			Multivariable*		
**Survival from Cancer Diagnosis Date**	**Hazard Ratio**	**95% CI**	**p-value**	**Hazard Ratio**	**95% CI**	**p-value**
CRP (time dependent)^¥^	1.005	(1.005, 1.006)	< .0001	1.003	(1.003, 1.004)	< .0001
Maximum CRP (continuous)	1.004	(1.004, 1.005)	< .0001	1.001	(1.000, 1.002)	0.005
Maximum CRP (categorized: high v normal)	2.76	(2.44, 3.11)	< .0001	1.57	(1.34, 1.84)	< .0001
TWBC (time dependent)^§^	1.06	(1.05, 1.07)	< .0001	1.02	(1.004, 1.04)	0.01
Maximum TWBC (continuous)	1.06	(1.05, 1.07)	< .0001	1.01	(0.998, 1.02)	0.10
Modified Glasgow Prognostic Score (mGPS)**						
Maximum CRP ≤ 10 mg/L	ref			ref		
Maximum CRP > 10 mg/L & Albumin ≥ 35 g/L	1.68	(1.44, 1.95)	< .0001	1.43	(1.22, 1.68)	< .0001
Maximum CRP > 10 mg/L & Albumin < 35 g/L	4.71	(4.14, 5.36)	< .0001	2.42	(2.06, 2.85)	< .0001
¥ 10mg/L u = increase § K/μL increase						
	**Univariable**			Multivariable*		
**Survival from Maximum CRP Test Date**	**Hazard Ratio**	**95% CI**	**p-value**	**Hazard Ratio**	**95% CI**	**p-value**
CRP (time dependent)^¥^	1.003	(1.002, 1.004)	< .0001	1.001	(1.000, 1.003)	0.03
Maximum CRP (continuous)	1.004	(1.004, 1.005)	< .0001	1.001	(1.000, 1.002)	0.005
Maximum CRP (categorized: high v normal)	2.65	(2.35, 3.00)	< .0001	1.50	(1.23, 1.76)	< .0001
TWBC (time dependent)^§^	1.02	(0.99, 1.05)	0.08	1.00	(0.97, 1.03)	0.98
Maximum TWBC (continuous)	1.05	(1.04, 1.06)	< .0001	1.01	(0.999, 1.02)	0.07
modified Glasgow Prognostic Score (mGPS)**						
CRP ≤ 10 mg/L	ref		< .0001	ref		< .0001
CRP > 10 mg/L & Albumin ≥ 35 g/L	1.69	(1.46, 1.97)		1.39	(1.18, 1.63)	
CRP > 10 mg/L & Albumin < 35 g/L	4.18	(3.68, 4.76)		2.31	(1.96, 2.71)	

^¥^ 10mg/L u = increase

^§^ K/μL increase

* Each multivariable model adjusted for the following: patient age at diagnosis, patient gender, WBC or CRP, hemoglobin, albumin, BMI, primary cancer site (respiratory, genital, digestive, urinary, breast, brain), liver metastases, number of metastatic sites, comorbidities (liver, cardiac, inflammatory bowel disease, rheumatic arthritis, venous thromboembolism)

** CRP and albumin removed from multivariable model to avoid multicollinearity

**Fig 3:**
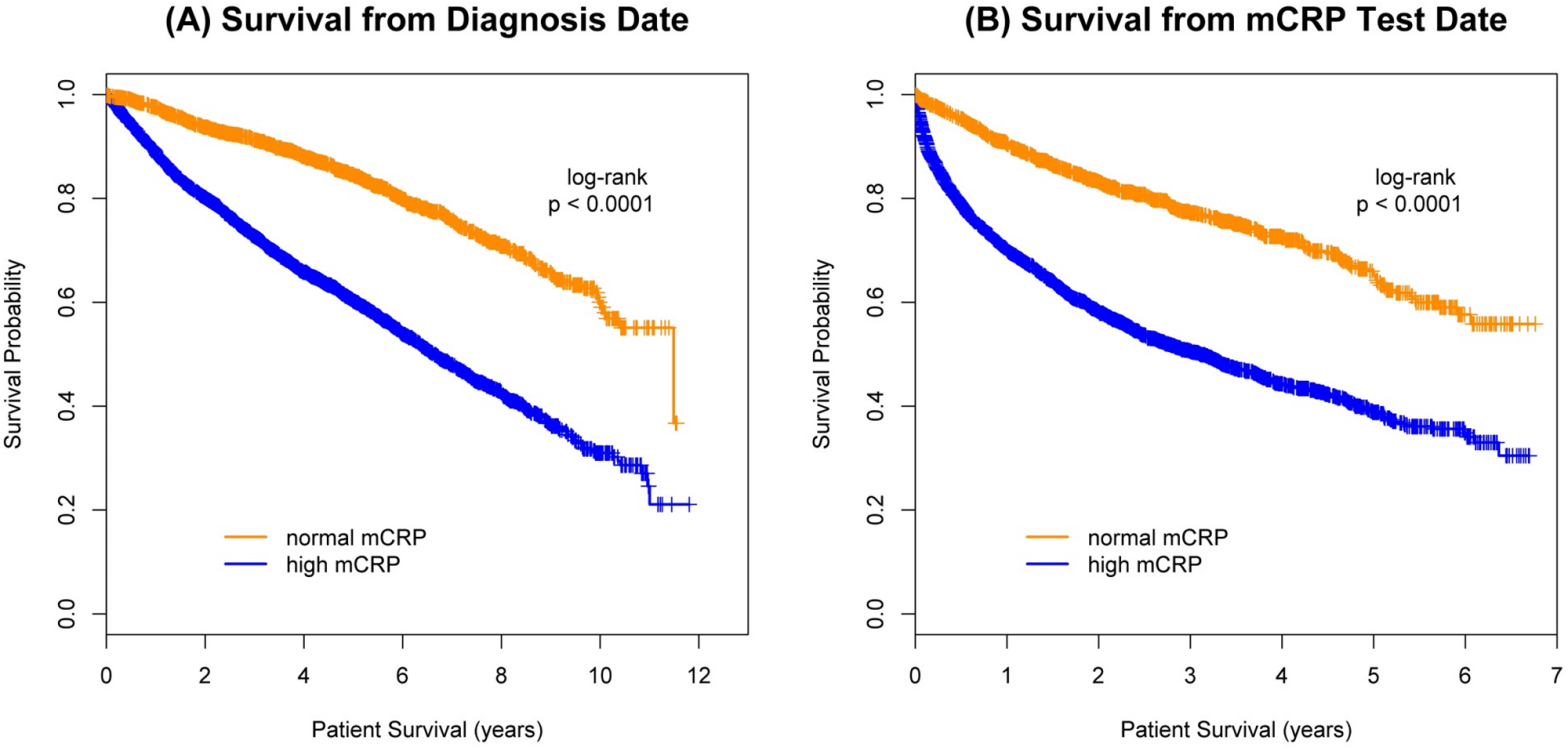
Prognosis by maximum CRP group.

Prognosis was analyzed by mCRP group (Fig 3). A significant survival difference was observed between the normal and high groups both from the date of cancer diagnosis (p < .0001; Fig 3A) and mCRP test date (p < .0001; Fig 3B). The risk of death increased 46% when mCRP was high (> 10 mg/L) compared to normal (≤ 10 mg/L) (HR 1.46; 95% CI 1.25–1.71; p < .0001) after control for all other model variables. A similar survival analysis was done from mCRP test date (Table 3). When the analyses were repeated, adjusting for time from cancer diagnosis, results did not change (Table 3). In both adjusted and unadjusted models, (regardless of how mCRP was analyzed), higher levels were associated with shorter survival, both from date of diagnosis and from date of test.

To further elucidate the mCRP association with survival, both the normal and high mCRP groups were subcategorized (Fig 1). In general, survival times were shorter in successive categories with higher mCRP values in both groups. The same analysis was performed from mCRP test day with similar results. When stratified by primary cancer site, higher mCRP values were associated with increased risk of death, regardless of tumor site. With mCRP as a time dependent variable, the hazard ratios (Δ = 10 mg/L) ranged from 1.05 to 1.13 (all p < .0001). When mCRP was categorized the hazard ratio for high compared to normal mCRP ranged from 1.35 to 7.37. The combination of low albumin and high mCRP in the (modified) Glasgow Prognostic Score supported these observations. Compared to the normal mCRP group, mGPS = 1 was associated with a 35% higher death risk whereas mGPS = 2 had an almost 250% increased risk. While not as strongly predictive, a higher TWBC was also associated with increased mortality. For each unit (k/μL) increase in TWBC, the risk of death rose 2% (HR 1.02; 95% CI 1.004–1.04; p = 0.01).

## Discussion

Two thirds of the large cohort of consecutive solid tumor patients who met the inclusion criteria had a high mCRP. This was consistently associated with shorter survival and was a powerful prognostic predictor in most solid tumors, regardless of statistical technique. The differences observed were both statistically and clinically important. When mCRP values above 10 mg/L were subcategorized, a higher mCRP was always worse. This approximate dose-response relationship provided further biological plausibility to the overall findings. Risk of early death also increased with high mCRP irrespective of cancer site. The highest mCRP values were noted among cancers of the esophagus, colon-rectum, bladder, and pancreas. This was the largest study of this issue to date. The total patient population was representative in terms of gender, ethnicity, and cancer primary sites. CRP seemed underused as a prognostic indicator as just two thirds met the inclusion criteria and most of those had few tests.

There were more males in the high mCRP group. Males with advanced cancers are known to lose more weight and have shorter survival than females [16]. High CRP may be positive and low albumin a negative marker of inflammation and loss of lean tissue mass. Lower hemoglobin and albumin levels were also noted amongst the high mCRP group. The hemoglobin and albumin abnormalities might be due to the generalized nutritional and functional decline common in cancer and/or reflect the secondary effects of a high inflammatory load.

Even among those with normal values, statistically and clinically significant shorter survival was noted at mCRP levels >5 mg/L. This suggests the need for a revised CRP reference range of 1–5 mg/L for prognostication in cancer patients. Few studies [17] have investigated this and it needs further research. Our results support the importance of CRP as a cost-effective independent prognostic indicator in most solid tumors. In addition, the scale and magnitude of the relationship suggest CRP should be recommended for routine use from diagnosis.

A relationship between inflammation and the origin of cancer was first hypothesized in 1863 [18]. This is now accepted but incompletely understood. Immune modulators have therapeutic benefit in specific tumors [19]. Some have advocated non-steroidal anti-inflammatory drugs (NSAIDs) in chemo-prevention and treatment and limited epidemiological evidence supports this [20]. NSAIDs (or corticosteroids, other immunomodulators) might be used as an adjuvant therapy to individualize therapy for solid tumor in those identified by high CRP values. The potential effectiveness of NSAIDs to reduce tumor recurrence, improve treatment response and reduce chemotherapeutic treatment-related toxicities needs further investigation.

This was a retrospective study. The original indication for the CRP measurement was not known that include nor was it part of any known research protocol or clinical pathway in that time frame. Some had multiple tests and others few. Currently, cancer prognosis is often (and usually inaccurately) estimated by clinical data like disease stage, performance status and histology. We found the EMR to be surprisingly unreliable regarding these characteristics and therefore were unable to incorporate them into the current analyses. Nonetheless, CRP was a highly significant independent predictor of survival from both diagnosis and test dates after adjustment for multiple other clinical variables (e.g. liver metastases) and exclusion of those potentially at risk for intercurrent infections.

Although adjustment for such additional covariates might have impacted some of our results, the sheer strength of the intimate association between high CRP and poor prognosis across multiple solid tumor primary sites makes this unlikely. Additionally, the strong relationship was observed even after control of multiple other covariates (that were available in the EMR). The available covariates e.g. liver metastases may also be seen as proxies for the information we could not access, like tumor histology. It is also noteworthy that the current use of parameters like performance status has not satisfactorily resolved the everyday prognostication clinical dilemma. Our results support the hypothesis that inflammation has an important role in cancer natural history and prognosis.

We believe our observations and conclusions are robust. We conducted a prior systematic review which found CRP to be prognostic of survival and treatment response specifically in gastrointestinal and renal cell carcinomas [21]. Our findings support these earlier observations [21, 22] but extend them to nearly all solid tumors. Some have proposed that tumoral CRP is superior to serum CRP for estimation of recurrence and prognosis [2, 23]. Others consider the CRP gene a potential target for individual therapy [24]. Polymorphism may increase cancer risk and has been associated with worse survival in colorectal cancer [25]. A relationship has also been proposed between systemic inflammation and various cancer symptoms. It appears that even one elevated CRP in a cancer patient (unexplained by a co-morbid illness or other intercurrent event) suggests a significantly worse disease outcome. Our data also supports the role of the GPS (although it might be enhanced by addition of TWBC).

Serial measurements to establish CRP kinetics may be clinically useful in different solid tumors and perhaps hematological malignancies and predict clinical course, cancer recurrence and survival [18]. CRP levels in both serum and tumor tissue could be evaluated among various solid tumors for prognostic purposes [23]. The relationship of CRP levels to specific histologies should also be investigated. Serum and tumoral CRP may also help target individualized therapy [2]. High sensitivity CRP (hsCRP), tumor CRP and CRP gene polymorphism should be evaluated for better insight into risk of recurrence, treatment response and toxicity [13, 23, 26]. Given the known close relationship between a high CRP and risk of cardiovascular disease, it is possible that accelerated atheromatous disease is part of the metastatic process and accompanies progressive disease. Lastly, it is possible that unexplained high CRP levels in otherwise healthy people may indicate a later risk of developing cancer (and not just cardiovascular disease). A recent systematic review and meta-analysis supports our observations [27]. CRP should be routinely used as a prognostic indicator in solid tumor oncology practice.

## Conclusions

Two thirds of the tested solid tumor population had a high mCRP. This was more common in males. In this representative cohort of consecutive solid tumor patients, the risk of death was clinically and statistically significantly greater for those with a high (or high normal) mCRP level independent of all other variables from both cancer diagnosis and test date. This was true regardless of primary cancer site and after exclusion of those with concurrent elevated white cell counts. Particular diseases (esophagus, colon-rectum, bladder, and pancreas) were more often associated with higher values. Risk of death was 46% greater when mCRP was high compared to normal. With each ten unit (mg/dl) increase the risk of increased 3%. There was also an inverse relationship between absolute mCRP value and survival even within the normal reference range. Lower serum albumin and hemoglobin levels were also noted among the high mCRP group. CRP seemed underutilized as a prognostic marker. Further analysis of the data set by the modified Glasgow Prognostic Score supported our observations. CRP should be used routinely in medical oncology practice to improve prognostic accuracy and further research is warranted in this important area.

## Supporting information

S1 File CRP DATA. (XLSX)
